# Shoulder Abduction While Using the Bougie: A Common Mistake

**DOI:** 10.5811/cpcem.2022.10.56372

**Published:** 2022-12-29

**Authors:** John J. Horky, Andrew P. Pirotte, Bailee R. Wilson

**Affiliations:** * University of Kansas Medical Center, Department of Emergency Medicine, Kansas City, Kansas; † University of Kansas School of Medicine, Kansas City, Kansas

**Keywords:** airway, bougie, intubation, emergency medicine

## Abstract

**Case Presentation:**

A 72-year-old female presented to the emergency department (ED) with exacerbation of chronic obstructive pulmonary disease and congestive heart failure. The patient required intubation for airway protection and hypercapnic respiratory failure. The ED team used a video laryngoscope, Macintosh 3 blade and bougie as the endotracheal tube delivery device. Despite a grade 2a Cormack-Lehane airway view, the bougie repeatedly missed left posterolateral to the airway. During these missed attempts, the emergency medicine (EM) resident’s shoulder was noted to be abducted. The EM resident then readjusted his technique by adducting the shoulder. which allowed the tip of the bougie to pass the vocal cords resulting in successful intubation.

**Discussion:**

The bougie is a useful endotracheal tube delivery device when used properly. Optimal body mechanics and device orientation are critical to successful use. Shoulder abduction while using the bougie is a frequent mistake, which can lead to left posterolateral malposition in relation to the glottis/airway. In this brief review our goal is to aid the intubating clinician in optimal use of the bougie, yielding more successful endotracheal tube passage.

## CASE PRESENTATION

A 72-year-old female with past medical history of severe chronic obstructive pulmonary disease (COPD) and congestive heart failure (CHF) presented to the emergency department with progressive shortness of breath. On examination, the patient was in respiratory distress and was encephalopathic. Initial venous blood gas confirmed hypercapnic respiratory failure with a pH of 7.21 (reference range: 7.35–7.44) and partial pressure of carbon dioxide (PCO_2_) of 102 millimeters of mercury (mm Hg) (36–50 mm Hg). Chest radiograph showed acute pulmonary edema. The patient was placed on bilevel positive airway pressure for a likely combined COPD and CHF exacerbation. Unfortunately, her mental status worsened, and repeat venous blood gas showed a pH of 7.12 and PCO_2_ of 120 mm Hg.

**Image 1:**
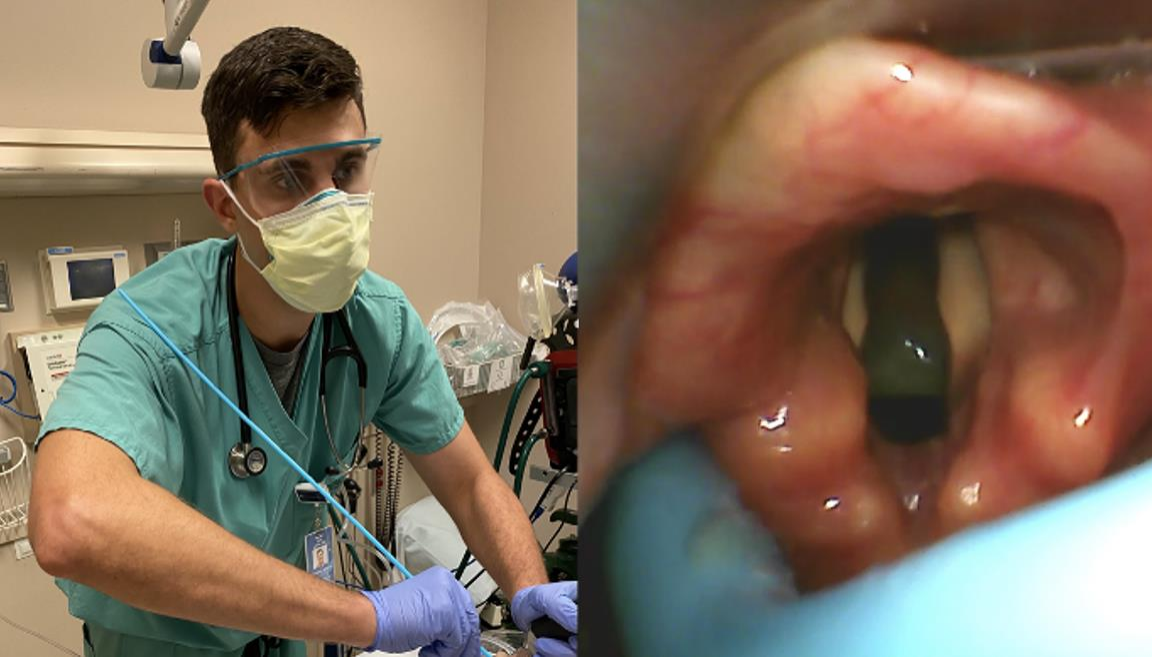
Emergency medicine resident’s right shoulder abducted correlating with the bougie missing left posterolateral to the airway.

**Image 2:**
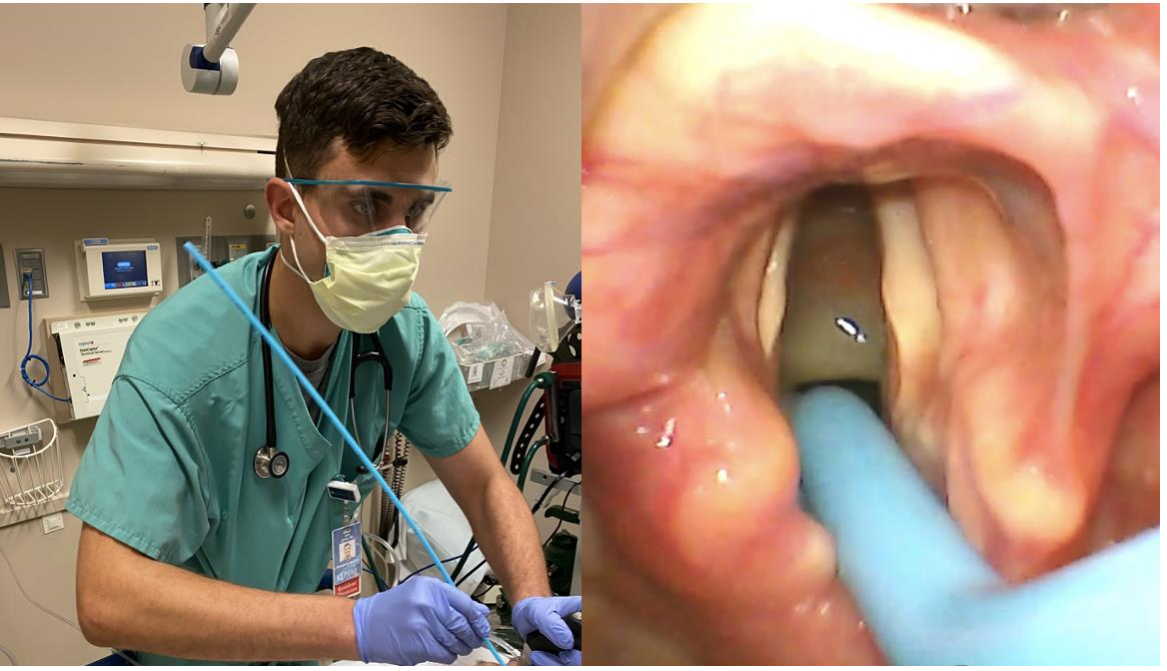
Emergency medicine resident’s right shoulder now adducted, which repositioned the tip of the bougie allowing it to pass through vocal cords.

The decision was made to intubate the patient for airway protection and hypercapnic respiratory failure. The ED team used a video laryngoscope, Macintosh 3 blade, and bougie as the endotracheal tube delivery device. Rapid sequence intubation was initiated with 150 milligrams (mg) of intravenous (IV) ketamine and 100 mg of IV rocuronium (patient weighed 86 kilograms). Despite a grade 2a Cormack-Lehane airway view, the bougie repeatedly missed left posterolateral to the airway (Image 1). During these missed attempts, the emergency medicine (EM) resident’s shoulder was noted to be abducted. The resident then readjusted his technique by adducting the shoulder, which allowed the tip of the bougie to pass the vocal cords resulting in successful intubation (Image 2).

## DISCUSSION

When the bougie is used as an adjunct with Macintosh video laryngoscopy in the ED, first-pass success has been reported at 98%.^1^ A survey of EM residency program directors found that the teaching and utilization of the bougie as an airway adjunct is rare.^2^ Unfamiliarity with the bougie leads to improper technique resulting in failure and abandonment of the bougie for other airway approaches. First-pass success for all other intubating approaches has been reported at 84%.^3^ Although shoulder mechanics while using the bougie has not been tracked or researched in any formal fashion, learners who abduct their shoulder resulting in the tip of the bougie missing the airway left posterolateral has been a frequently observed behavior in our ED over the past several years.

While our patient did not decompensate during missed attempts (ongoing apneic oxygenation likely prevented any desaturation), patient deterioration from a delay in intubation by improper technique is a risk.

Shoulder abduction (i.e., “chicken-winging”) results in an oblique orientation of the bougie relative to the airway. Shoulder adduction (tucking elbow to side) results in parallel orientation of the bougie and airway. It is important to note that all clinicians use the bougie differently and this technique may not apply to all intubators. For example, some bougie users will pre-load the endotracheal tube onto the bougie, and shoulder mechanics may affect a preloaded bougie differently. As use of the bougie becomes more common, there is value in dedicated instruction on proper technique and use of the device.

*CPC-EM Capsule*

What do we already know about this clinical entity?

*Using the bougie with proper technique as an airway adjunct with Macintosh video laryngoscopy has a high first-pass success in the emergency department.*

What is the major impact of the image(s)?

*If the clinician tucks their elbow to the side (shoulder adduction), this parallel orientation of the bougie and airway increases successful use of the device.*

How might this improve emergency medicine practice?

*By emphasizing proper bougie technique in medical education, clinicians may improve the rate of successful intubation.*
